# Low serum pseudocholinesterase levels are associated with mortality in patients with hepatocellular carcinoma

**DOI:** 10.1097/HC9.0000000000000879

**Published:** 2026-01-05

**Authors:** Karina Sato-Espinoza, Marie-Lise Chrysostome, Robert A. Vierkant, Hunter B. Miller, Perapa Chotiprasidhi, Sumera I. Ilyas, Lewis R. Roberts, Kirk J. Wangensteen

**Affiliations:** 1 Department of Medicine, Division of Gastroenterology, Mayo Clinic, Rochester, Minnesota, USA; 2 Department of Quantitative Health Sciences, Division of Biomedical Statistics and Informatics, Mayo Clinic, Rochester, Minnesota, USA

**Keywords:** hepatectomy, hepatitis C virus, hepatocellular carcinoma, prognosis, pseudocholinesterase

## Abstract

**Background:**

There is no consensus scoring system for staging and prognosis in hepatocellular carcinoma (HCC), which is the fourth leading cause of cancer-related mortality worldwide. Commonly used systems include the albumin–bilirubin (ALBI) score, the Barcelona staging classification (Barcelona Clinic Liver Cancer, BCLC), the Model for End-Stage Liver Disease (MELD), and the model to estimate survival in ambulatory HCC patients (MESIAH). Liver secretion of pseudocholinesterase (PCHE) has been linked to liver function but is poorly studied in the natural history of HCC. We evaluated whether serum PCHE level predicts HCC mortality and whether it enhances existing scoring systems.

**Methods:**

We conducted a retrospective cohort study of individuals diagnosed with HCC. We collected variables including PCHE level, clinical data used in scoring systems, and time to mortality or liver transplant. We then analyzed the association between these variables and survival using Kaplan–Meier curves, Cox proportional hazards regression models, receiver operating characteristic (ROC) curves, and area under the curve (AUC) calculations.

**Results:**

We identified 420 individuals with HCC who were tested for PCHE levels, with a follow-up time of more than 20 years. There was a strong inverse relationship between PCHE level and overall survival, with the lowest quartile having high mortality and poor outcomes. Low PCHE level was associated with hepatitis C virus (HCV) infection, vascular invasion, poor liver function, and a high likelihood of liver transplant. In contrast, the highest quartile was associated with metabolic dysfunction–associated steatotic liver disease (MASLD) as the underlying cause. Compared with validated scoring systems, ALBI, BCLC, MELD 3.0, and MESIAH, the PCHE level was an independent predictor of mortality. PCHE levels could predict 3-month survival as well as or better than the other scoring systems, with an AUC of 0.74. PCHE level could also predict mortality related to hepatectomy. The addition of PCHE level to MESIAH and ALBI scoring systems could further improve the ability to predict overall mortality in HCC.

**Conclusions:**

PCHE is a reliable stand-alone biomarker of HCC prognosis. It is an independent predictor of mortality and can improve the accuracy of existing scoring systems. Improved risk stratification could improve outcomes by informing treatment decisions regarding hepatectomy or other interventions.

## Graphical abstract

**Figure:**
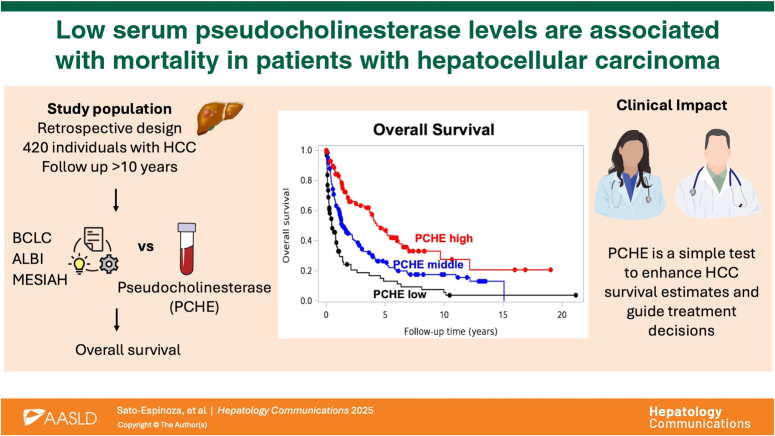

## INTRODUCTION

Liver cancer is the sixth most common cancer worldwide and is a significant cause of cancer deaths in the United States.^1^ Hepatocellular carcinoma (HCC) accounts for ~80% of cases of primary liver cancer.^2^ HCC develops in individuals with cirrhosis from diverse etiologies such as viral hepatitis, alcohol consumption, or metabolic dysfunction–associated steatotic liver disease (MASLD).^3^,^4^ Liver failure is the most common cause of death in patients with HCC.^5^ There is often replacement of functional liver tissue with cancer.^6^,^7^ In addition, the tumor can obstruct blood flow, particularly the portal system, leading to further complications such as gastrointestinal bleeding, sepsis, and renal failure, which can be fatal.^8^,^9^ Predicting the prognosis remains challenging due to the complexity and heterogeneity of HCC and the absence of standardized biomarkers that accurately forecast the prognosis.

The traditional tumor–node–metastasis (TNM) staging system, often used for other types of solid tumors, is not very effective for prognosis and staging in HCC.^10^ Instead, a variety of alternative staging and prognostic scores have been proposed and validated. The most widely used systems are: (1) The albumin–bilirubin (ALBI) score, which also evaluates liver function and serves as a prognostic indicator for patients with HCC.^11^ This score can help predict overall survival (OS) regardless of the treatment modality. (2) The Barcelona staging classification (Barcelona Clinic Liver Cancer, BCLC), which assesses tumor burden, liver function, and physical status.^12^,^13^ This staging system provides a comprehensive overview of the disease, which helps guide therapeutic decisions in daily clinical practice. It is also used to estimate the survival. (3) The Model for End-stage Liver Disease (MELD) version 3.0 serves as a mortality prediction tool for all causes of advanced liver diseases.^14^ (4) The model to estimate survival in ambulatory HCC patients (MESIAH) score evaluates tumor characteristics and liver function. This score predicts OS in individuals with HCC, regardless of the type of treatment or underlying causes of liver disease.^15^ Although diverse score systems are available, none have achieved universal adoption, highlighting the continued need for refinement and standardization of HCC prognostic and staging methodologies.

Pseudocholinesterase (hereafter abbreviated PCHE), also referred to as butyrylcholinesterase, is encoded by *BCHE*. It is an alpha-glycoprotein that is highly expressed by hepatocytes and is secreted into the blood.^16^ The protein has been studied as an independent risk factor for various cancers; the association with cancer is inverse to the expression level of PCHE for bladder,^17^ pancreas,^18^ cervix,^19^ colorectal,^20^ and prostate^21^ cancers. The level is also inversely associated with the severity of liver diseases,^22^,^23^ inflammation, and protein-energy malnutrition.^24^ However, there is only limited evidence in the literature regarding the association between PCHE and mortality from HCC.

At our institution, the PCHE test is part of a liver function assessment template for patients. This test was used for many patients with HCC, even though there were no specific guidelines on its utility as a biomarker in HCC.

We aimed to assess the value of serum PCHE levels as a prognostic biomarker in individuals with HCC. We hypothesized that since PCHE is mainly expressed by hepatocytes, it could reflect liver functional reserve, and therefore the fitness of patients who have HCC. We included patients of all etiological backgrounds and treatment modalities. In addition, we sought to compare the predictive effectiveness of PCHE levels to determine whether it could enhance the established scoring systems ALBI,^11^ BCLC stage system,^13^ MELD 3.0,^14^ and MESIAH.^15^

## METHODOLOGY

We conducted a retrospective cohort study of individuals diagnosed with HCC at a single tertiary care center, the Mayo Clinic. Our study was conducted in accordance with both the Declarations of Helsinki and Istanbul. The Mayo Clinical Institutional Review Board (IRB) approved the study under Protocol No. 707-03. HCC diagnosis was based on radiological images or biopsy results according to AASLD guidelines.^25^ Data on comorbidities, imaging, and biochemical tests were collected from the electronic health record (EHR) system.

The PCHE value and the score for ALBI, MELD 3.0, and MESIAH were categorized into the following 3 tiers based on their observed distributions in our dataset to examine associations at the highest and lowest values of the ranges: the lowest 25th percentile (Q1), the middle 50th percentile (Q2–Q3), and the highest 25th percentile (Q4). BCLC stage was categorized as 0/A, B, C, and D.

Data were summarized using frequencies and percentages for categorical variables, and medians and ranges for continuous variables. Associations of categorized PCHE values with demographic and clinical characteristics were assessed using analyses of variance for continuous variables and chi-square tests or, if any of the expected counts were <5, the Fisher exact tests for categorical variables. Associations of continuously distributed PCHE with MESIAH, ALBI, MELD 3.0, and BCLC stage were assessed using Pearson correlation coefficients. For the latter, BCLC was treated as an ordinal effect.

Patients were followed from the date of the PCHE lab draw to the date of death or date of last follow-up, with additional right censorship at the date of transplant for those undergoing liver transplants. Kaplan–Meier curves and corresponding log-rank tests examined associations of OS with the categorized PCHE or the scoring system of interest. Cox proportional hazards regression analyses formally assessed associations of these same measures with risk of death. Two sets of analyses were carried out: a series of univariable analyses that examined each score in turn with risk of death, and a multivariable analysis that simultaneously included all 5 scores as exposure variables in the same model to assess the independent effects of each on risk of death. Models were first fit to examine associations with categorized scores, using the following as referent groups: PCHE, Q4; MESIAH, Q1; ALBI, Q1; MELD 3.0, Q1; BCLC, stage 0/A. These were followed by analyses that examined dose–response associations with risk of death by fitting the scores as continuous effects. For these models, the raw PCHE, MESIAH, ALBI, and MELD 3.0 score values were divided by the standard deviation (SD) of their values, such that hazard ratios can be interpreted as the change in the risk of death per 1 SD increase in the score of interest.

We assessed the ability of the continuously or ordinally distributed liver function scores, including PCHE, to discriminate between patients who did and did not die within 3 months of the lab test using logistic regression models to generate receiver operator characteristic (ROC) curves. Discrimination was summarized by calculating the area under each curve (AUC, or alternatively, concordance index) along with 95% confidence intervals. Separate logistic models were first fit for each of the 5 liver function scores. These were followed by another series of 4 logistic models that fit PCHE plus 1 of the other liver function scores in the same model to assess the change in AUC.

## RESULTS

### Pseudocholinesterase level is inversely associated with markers of liver reserve function, tumor vascular invasion, and overall survival

We identified 420 individuals with HCC and a PCHE level. The study population was predominantly male (77.9%), with a median age of 65.5 years. Most individuals had cirrhosis at baseline (89.5%).

Based on our hypothesis that PCHE levels are correlated with liver synthetic functions, we subdivided the individuals into a high-risk, low expression tier (Q1, 105 individuals, 25%), a moderate-risk, medium expression tier (Q2–Q3, 210 individuals, 50%), and a low-risk, high expression tier (Q4, 105 individuals, 25%).

The underlying causes of liver disease varied significantly across risk tiers (Table 1). The most common etiology in Q1 was hepatitis C virus (HCV) (56.2%). In Q2–Q3, HCV and MASLD shared the top position (25.7% each). The predominant etiology in Q4 was MASLD (42.9%).

**TABLE 1:** Associations of categorized pseudocholinesterase values with demographic and clinical characteristics

	Pseudocholinesterase per upper limit of normal				
	Q1 (N=105)	Q2–Q3 (N=210)	Q4 (N=105)	Total (N=420)	*p*
Age at lab test					<0.001^a^
N	105	210	105	420	
Median (range)	60.2 (38.2, 81.5)	66.7 (23.8, 92.5)	68.3 (32.4, 93.3)	65.5 (23.8, 93.3)	
Sex, n (%)					0.011^b^
Female	17 (16.2%)	42 (20.0%)	34 (32.4%)	93 (22.1%)	
Male	88 (83.8%)	168 (80.0%)	71 (67.6%)	327 (77.9%)	
Cirrhosis, n (%)					0.296^b^
No cirrhosis	7 (6.7%)	26 (12.4%)	11 (10.5%)	44 (10.5%)	
Cirrhosis	98 (93.3%)	184 (87.6%)	94 (89.5%)	376 (89.5%)	
Etiology, n (%)					<0.001^c^
ALD	13 (12.4%)	19 (9.0%)	4 (3.8%)	36 (8.6%)	
Cryptogenic	11 (10.5%)	53 (25.2%)	22 (21.0%)	86 (20.5%)	
Hemochromatosis	2 (1.9%)	9 (4.3%)	5 (4.8%)	16 (3.8%)	
HBV	1 (1.0%)	15 (7.1%)	5 (4.8%)	21 (5.0%)	
HCV	59 (56.2%)	54 (25.7%)	21 (20.0%)	134 (31.9%)	
MASLD	15 (14.3%)	54 (25.7%)	45 (42.9%)	114 (27.1%)	
Other	4 (3.8%)	6 (2.9%)	3 (2.9%)	13 (3.1%)	
Treatment received, n (%)					<0.001^c^
Locoregional therapy	54 (51.4%)	111 (52.9%)	53 (50.5%)	218 (51.9%)	
Hepatectomy	2 (1.9%)	29 (13.8%)	33 (31.4%)	64 (15.2%)	
Systemic therapy	6 (5.7%)	20 (9.5%)	6 (5.7%)	32 (7.6%)	
Liver transplantation	6 (5.7%)	10 (4.8%)	3 (2.9%)	19 (4.5%)	
Palliative care	37 (35.2%)	36 (17.1%)	7 (6.7%)	80 (19.0%)	
Other therapy	0 (0.0%)	4 (1.9%)	3 (2.9%)	7 (1.7%)	
Tumor number, n (%)					0.775^c^
1	59 (78.7%)	135 (81.3%)	74 (78.7%)	268 (80.0%)	
2	9 (12.0%)	14 (8.4%)	12 (12.8%)	35 (10.4%)	
3	3 (4.0%)	12 (7.2%)	5 (5.3%)	20 (6.0%)	
4 or more	4 (5.3%)	5 (3.0%)	3 (3.2%)	12 (3.6%)	
Missing	30	44	11	85	
Maximum tumor size					0.570^a^
N	66	157	94	317	
Median (range)	4.1 (1.0, 20.0)	5.1 (0.2, 114.4)	5.1 (0.9, 33.0)	4.8 (0.2, 114.4)	
Metastasis, n (%)					0.621^b^
No metastasis	74 (85.1%)	162 (88.5%)	85 (89.5%)	321 (87.9%)	
Metastasis	13 (14.9%)	21 (11.5%)	10 (10.5%)	44 (12.1%)	
Missing	18	27	10	55	
AFP (ng/mL)					0.408^a^
N	95	182	99	376	
Median (range)	98.0 (0.8, 378,160.0)	24.0 (0.7, 278,567.0)	7.1 (0.8, 165,501.0)	28.0 (0.7, 378,160.0)	
DCP (ng/mL)					0.495^a^
N	9	43	31	83	
Median (range)	74.0 (0.3, 1910.0)	23.0 (0.2, 19,882.0)	36.0 (0.3, 3348.0)	36.0 (0.2, 19,882.0)	
AFP-L3 (%)					0.735^a^
N	10	44	32	86	
Median (range)	22.5 (0.5, 60.0)	20.5 (0.3, 89.0)	9.4 (0.5, 85.9)	18.5 (0.3, 89.0)	
Albumin (g/dL)					<0.001^a^
N	105	210	105	420	
Median (range)	3.1 (2.1, 4.6)	3.6 (2.3, 4.6)	4.2 (2.4, 4.9)	3.6 (2.1, 4.9)	
Bilirubin (mg/dL)					<0.001^a^
N	105	210	104	419	
Median (range)	2.0 (0.2, 25.5)	1.0 (0.1, 22.9)	0.6 (0.2, 4.5)	1.0 (0.1, 25.5)	
Platelets (×10^9^/L)					<0.001^a^
N	101	204	103	408	
Median (range)	120.0 (18.0, 559.0)	148.5 (32.0, 781.0)	206.0 (26.0, 485.0)	153.5 (18.0, 781.0)	
Creatinine (mg/dL)					0.130^a^
N	105	210	105	420	
Median (range)	0.9 (0.5, 8.2)	0.9 (0.3, 3.3)	1.0 (0.5, 2.0)	1.0 (0.3, 8.2)	
INR					0.018^a^
N	105	210	105	420	
Median (range)	1.2 (0.2, 2.6)	1.1 (0.9, 7.5)	1.0 (0.8, 2.8)	1.1 (0.2, 7.5)	
Sodium (mmol/Eq)					<0.001^a^
N	105	210	105	420	
Median (range)	137.0 (124.0, 144.0)	140.0 (124.0, 150.0)	140.0 (133.0, 145.0)	140.0 (124.0, 150.0)	
CRP (mg/dL)					0.623^a^
N	3	6	9	18	
Median (range)	9.6 (2.0, 10.9)	3.2 (0.9, 6.9)	0.8 (0.2, 24.8)	1.3 (0.2, 24.8)	
Sed rate (mm/h)					0.491^a^
N	5	5	5	15	
Median (range)	45.0 (5.0, 59.0)	29.0 (9.0, 135.0)	27.0 (6.0, 65.0)	29.0 (5.0, 135.0)	
Child–Pugh grade, n (%)					<0.001^b^
A	14 (13.3%)	84 (40.0%)	79 (75.2%)	177 (42.1%)	
B	53 (50.5%)	110 (52.4%)	25 (23.8%)	188 (44.8%)	
C	38 (36.2%)	16 (7.6%)	1 (1.0%)	55 (13.1%)	
Vascular invasion, n (%)					0.005^b^
No	12 (34.3%)	47 (48.0%)	44 (66.7%)	103 (51.8%)	
Yes	23 (65.7%)	51 (52.0%)	22 (33.3%)	96 (48.2%)	
Missing	70	112	39	221	
ECOG status, n (%)					<0.001^b^
0	13 (12.5%)	54 (26.3%)	43 (41.3%)	110 (26.6%)	
1	44 (42.3%)	91 (44.4%)	40 (38.5%)	175 (42.4%)	
2	37 (35.6%)	43 (21.0%)	14 (13.5%)	94 (22.8%)	
3	7 (6.7%)	17 (8.3%)	6 (5.8%)	30 (7.3%)	
4	3 (2.9%)	0 (0.0%)	1 (1.0%)	4 (1.0%)	
Missing	1	5	1	7	
MELD version 3.0					<0.001^a^
N	103	199	91	393	
Median (range)	15.0 (6.0, 37.0)	10.0 (6.0, 29.0)	8.0 (6.0, 21.0)	10.0 (6.0, 37.0)	
BCLC stage, n (%)					<0.001^b^
0/A	9 (10.2%)	20 (12.3%)	9 (10.3%)	38 (11.2%)	
B	24 (27.3%)	77 (47.2%)	55 (63.2%)	156 (46.2%)	
C	17 (19.3%)	50 (30.7%)	22 (25.3%)	89 (26.3%)	
D	38 (43.2%)	16 (9.8%)	1 (1.1%)	55 (16.3%)	
Missing	17	47	18	82	
MESIAH score					0.021^a^
N	95	181	99	375	
Median (range)	4.6 (2.9, 7.4)	4.4 (2.2, 7.5)	4.2 (2.6, 6.8)	4.4 (2.2, 7.5)	
ALBI score					<0.001^a^
N	104	208	104	416	
Median (range)	−1.7 (−3.1, −0.3)	−2.3 (−3.4, −0.8)	−2.8 (−3.6, −1.3)	−2.3 (−3.6, −0.3)	

^a^ Analysis of variance.

^b^ Chi-square test.

^c^ Fisher exact test.

Abbreviations: AFP, alpha-fetoprotein; AFP-L3, alpha-fetoprotein L3 percent; ALBI, albumin–bilirubin; LD, alcoholic liver disease; BCLC, Barcelona Clinic Liver Cancer; CRP, C-reactive protein; ECOG, Eastern Cooperative Oncology Group performance status; HBV, hepatitis B virus; HBC, hepatitis C virus; INR, international normalized ratio; MASLD, metabolic dysfunction–associated steatotic liver disease; MELD, Model for End-Stage Liver Disease; MESIAH, model to estimate survival in ambulatory HCC patients; Sed, sedimentation.

In tier Q1, the predominant treatments administered were locoregional therapy (51.4%) and palliative care (35.2%), while systemic therapy and liver transplantation each accounted for 5.7% of cases. In the second tier, from Q2 to Q3, locoregional therapy remained the most frequently employed treatment, at 52.9%, followed by palliative care at 17.1% and hepatectomy at 13.8%. In the third tier, Q4, locoregional therapy again emerged as the most common intervention at 50.5%, with hepatectomy exhibiting a higher prevalence at 31.4% compared with the other tiers (Table 1).

Individuals in tier Q1 had a higher rate of vascular invasion compared with Q2–3 and Q4 (65.7% vs. 52% and 33.3%, *p*=0.005). Furthermore, tier Q1 exhibited the poorest baseline liver function compared with the other tiers, with lower levels of albumin and platelets and higher levels of bilirubin and INR. However, there was no statistically significant difference in tumor number, maximum tumor size, or metastases. Additional details are shown in Table 1.

**FIGURE 1:**
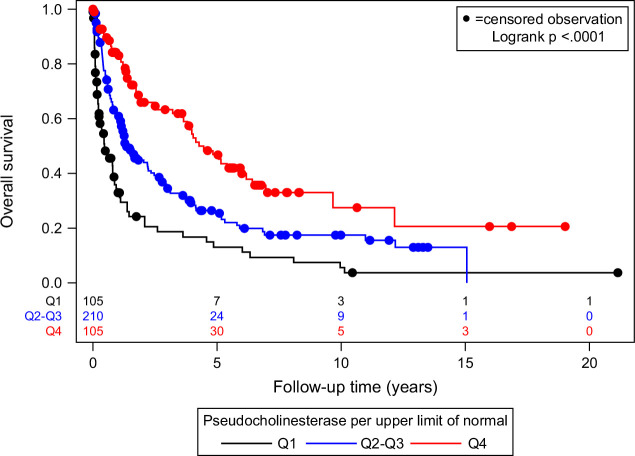
Association of categorized pseudocholinesterase with overall survival.

We analyzed OS in the PCHE tiers. We found a strong inverse relationship between the PCHE levels and the OS. Q1 had the poorest OS, followed by Q2–Q3 with an intermediate survival rate, and Q4 with the best OS (log-rank test *p*<0.0001, Figure 1, Table 2). This finding highlights the potential of PCHE levels as a prognostic marker for individuals with HCC.

**TABLE 2:** Univariable and multivariable associations of categorized pseudocholinesterase and other liver function scores with risk of death

Variable	# patients	# deaths	Person-years	HR (95% CI)^a^	*p* ^a^	HR (95% CI)^b^	*p* ^b^
Pseudocholinesterase					<0.001		<0.001
Q1	105	72	125.6	3.52 (2.45–5.05)		3.00 (1.75–5.17)	
Q2–Q3	210	127	423.8	1.82 (1.31–2.51)		1.38 (0.90–2.12)	
Q4	105	52	356.7	1.00 (ref)		1.00 (ref)	
MESIAH					<0.001		0.756
Q1	91	40	306.1	1.00 (ref)		1.00 (ref)	
Q2–Q3	188	117	379.3	1.97 (1.37–2.83)		1.13 (0.60–2.12)	
Q4	96	60	145.1	2.61 (1.74–3.91)		1.28 (0.63–2.59)	
ALBI					<0.001		0.544
Q1	102	52	332.7	1.00 (ref)		1.00 (ref)	
Q2–Q3	209	131	466.6	1.70 (1.23–2.35)		1.21 (0.77–1.92)	
Q4	105	68	93.3	3.67 (2.53–5.32)		1.01 (0.55–1.85)	
MELD 3.0					<0.001		0.042
Q1	125	69	388.3	1.00 (ref)		1.00 (ref)	
Q2–Q3	138	96	294.7	1.65 (1.21–2.25)		1.37 (0.91–2.05)	
Q4	130	80	161.6	2.43 (1.76–3.37)		1.85 (1.15–2.99)	
BCLC					<0.001		0.001
0/A	38	13	201.8	1.00 (ref)		1.00 (ref)	
B	156	102	357.6	3.38 (1.89–6.04)		4.63 (2.22–9.69)	
C	89	55	150.9	4.15 (2.25–7.64)		4.31 (1.92–9.71)	
D	55	30	32.0	7.59 (3.89–14.8)		4.87 (2.07–11.5)	

^a^ Univariable Cox proportional hazards regression model, fitting time to death as outcome and variable of interest as exposure.

^b^ Multivariable Cox proportional hazard regression model, fitting time to death as outcome and simultaneously including all 5 variables in the same model as exposure variables.

Abbreviations: ALBI, albumin–bilirubin; BCLC (Barcelona Clinic Liver Cancer), Barcelona staging system; CI, confidence interval; HR, hazard ratio; MELD, Model for End-Stage Liver Disease; MESIAH, model to estimate survival in ambulatory HCC patients.

We further analyzed whether PCHE levels predict survival in different subgroups of individuals with HCC. For the 44 patients with non-cirrhotic HCC, low PCHE level was associated with increased mortality with a HR of 3.86 (*p*=0.050). The same was true for the 134 patients with HCV (HR of 2.80, *p*<0.001) and the 114 with MASLD (HR of 4.50, *p*<0.001).

### Pseudocholinesterase level alone predicts mortality from HCC, as well as scoring systems

As expected based on previous reports, the level of MESIAH, ALBI, MELD 3.0, was correlated with mortality (log-rank tests *p*<0.001 for each, Supplemental Figure S1, http://links.lww.com/HC9/C210, Table 2). Specifically, tier Q1 demonstrated the best OS, while Q4 exhibited the lowest OS for these scoring systems. With the BCLC staging system, stage 0/A had the best OS, which gradually decreased for stages B and C, culminating in the lowest survival rates at stage D (Supplemental Figure S1, http://links.lww.com/HC9/C210).

We analyzed the relationship between PCHE levels and the scoring systems. We found PCHE levels are inversely correlated with MESIAH score (*r*=−0.15), ALBI score (*r*=−0.65), MELD 3.0 score (*r*=−0.41), and BCLC stage (*r*=−0.31) (Supplemental Figure S2, http://links.lww.com/HC9/C210).

We performed a multivariable analysis for the scoring systems, and a low PCHE level remained strongly associated with the risk of death with an HR of 3.00 for Q1 compared with Q4 (95% CI 1.75–5.17; *p*<0.001, Table 2). Notably, multivariable analysis yielded non-significant results in Q4 for MESIAH HR of 1.28 (95% CI 0.63–2.59) and Q4 for ALBI HR of 1.01 (95% CI 0.55–1.85) (Table 2). The significant association of PCHE with mortality risk, even after adjustment for the effects of the other scores, indicates it was an independent prognostic marker.

Overall, PCHE may serve as a valuable independent biomarker in assessing the risk of mortality in individuals with HCC.

### Pseudocholinesterase level discriminates 3-month survival as well as existing scoring systems

**FIGURE 2:**
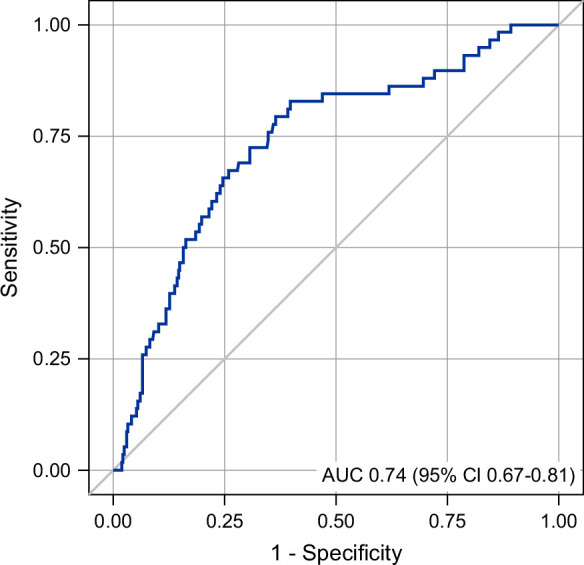
Association of pseudocholinesterase with 3-month survival, represented as an ROC curve. Abbreviations: AUC, area under the curve; ROC, receiver operating characteristic.

PCHE levels could discriminate 3-month survival with an AUC of 0.74 (95% CI 0.67–0.81) (Figure 2). The AUC of the scoring systems was 0.70 for MESIAH (95% CI 0.62–0.77), 0.74 for ALBI (95% CI 0.68–0.81), 0.73 for MELD 3.0 (95% CI 0.66–0.80), and 0.67 for BCLC (95% CI 0.60–0.75) (Supplemental Figure S3, http://links.lww.com/HC9/C210). PCHE alone had as high an AUC as any of the other scoring systems.

We further analyzed the ability of PCHE to estimate mortality by dividing the population into 10 equal groups (deciles) defined by PCHE values. The proportion of individuals who died within 3 months varied significantly across the deciles of PCHE values (*p*<0.001). For example, the mortality at 3 months was 36.6% for individuals in the first decile and only 2.4% in the tenth decile (Supplemental Table S1, http://links.lww.com/HC9/C211). These findings suggest that values at the extremes of PCHE continue to serve as valuable indicators of mortality rates.

### Addition of pseudocholinesterase to each individual scoring system improves performance

To assess whether PCHE contributes additional discriminative value to existing scoring systems, we evaluated predictive models that combined PCHE levels with other scoring systems. By including PCHE together with the scoring system in the logistic regression model, we were able to improve the AUC beyond the performance of each individual scoring system. The best performing model was the MESIAH score plus PCHE, in which the AUC increased to 0.78 (95% CI 0.72–0.84) (Table 3).

**TABLE 3:** Three-month survival model discrimination for pseudocholinesterase and other predictors using area under the ROC curve (AUC)

Predictors	N^a^	AUC (95% CI)^b^
Main effects models		
Pseudocholinesterase	420	0.74 (0.67–0.81)
MESIAH	375	0.70 (0.62–0.77)
ALBI	416	0.74 (0.68–0.81)
MELD	393	0.73 (0.66–0.80)
BCLC	338	0.67 (0.60–0.74)
Two-variable models		
Pseudocholinesterase + MESIAH	375	0.78 (0.72–0.84)
Pseudocholinesterase + ALBI	416	0.75 (0.69–0.81)
Pseudocholinesterase + MELD	393	0.75 (0.68–0.82)
Pseudocholinesterase + BCLC	338	0.74 (0.67–0.81)

^a^ Sample size varies due to missing values for variables of interest.

^b^ Logistic regression model, fitting vital status at 3 months post diagnosis as outcome, and all continuously distributed variables listed in the predictors column as exposures.

Abbreviations: ALBI, albumin–bilirubin; BCLC (Barcelona Clinic Liver Cancer), Barcelona staging system; CI, confidence interval; HR, hazard ratio; MELD, Model for End-Stage Liver Disease; MESIAH, model to estimate survival in ambulatory HCC patients.

## DISCUSSION

To our knowledge, this is the first study analyzing PCHE in individuals diagnosed with HCC undergoing various treatments, including locoregional therapy, hepatectomy, systemic therapy, and liver transplantation (Table 1). We found PCHE is an independent predictor of mortality for individuals with HCC, which provided additional prognostic value to existing scoring systems, particularly the MESIAH score.

We found that PCHE levels correlate with markers of liver function. The Q1 tier (high risk/low expression of PCHE) exhibited lower levels of albumin and platelets and higher levels of INR and bilirubin, indicating compromised liver function. Existing prognostic HCC scores include markers of liver function, but our analysis indicates that PCHE is not only correlated with markers of liver synthetic function but also has independent prognostic value and is informative across a broad range of levels. PCHE level also correlated with underlying liver disease etiology, with HCV more common in the higher risk quartile (Q1) and MASLD in the lower risk quartile (Q4), possibly reflective of different amounts of liver reserve according to the liver disease etiology. However, PCHE was accurate in predicting mortality in each of these different liver disease etiologies and for non-cirrhotic HCC.

PCHE has not only been found to be a predictor for cancer but has also been correlated to liver disease,^23^ inflammation, and protein-energy malnutrition.^24^ It exhibits similarities and differences in comparison to existing tests of liver health. PCHE, albumin, and clotting factors share the characteristic of being produced in the liver and serve as indicators of liver biosynthetic function.^26^,^27^,^28^,^29^ This is distinct from alanine aminotransferase and aspartate aminotransferase which denote liver injury, and alkaline phosphatase and bilirubin which primarily indicate cholestasis.^29^,^30^ While albumin levels typically decrease due to impaired synthesis in liver disease,^31^,^32^ PCHE levels decrease due to both impaired synthesis and reduced enzymatic activity.^24^,^33^ In an inflammatory state, albumin acts as a negative acute phase reactant as cytokines downregulate production in the liver.^34^,^35^ Evidence suggests PCHE may be impacted similarly, as decreased levels have been observed in inflammatory states.^36^,^37^,^38^,^39^ Several liver tests can also be impacted by extrahepatic processes. Protein wasting in nephrotic syndrome can deplete albumin, and drugs or clotting factor consumption can increase INR and prothrombin time. In cancer, low PCHE levels could result from underproduction, increased consumption, or enzyme degradation due to systemic inflammation and tumor metabolism.^18^ Furthermore, in a state of malnutrition, the availability of amino acids and overall protein synthesis are disrupted, influencing PCHE levels.^24^ More research is needed to determine how these mechanisms come together to inform PCHE’s metabolic profile.

PCHE has been studied in various cancers,^40^ such as bladder,^17^ pancreas,^18^ cervix,^19^ head and neck,^18^ colorectal,^20^ and prostate,^21^ where it has been shown to be prognostic. A recent study conducted on individuals with HCC evaluated PCHE levels plus albumin before surgery, and found that the levels predict perioperative mortality in individuals undergoing hepatectomy as a primary curative treatment.^41^ Our study identified 64 patients who underwent hepatectomy. In tier Q1, 2/2 patients died; in tier Q2–Q3, 18/29 (62%) died, while in tier Q4, 12/33 (36.4%) died (*p*<0.05, data not shown). These findings corroborate previous results reported by Tadokoro et al,^41^ which indicated that patients with low levels of PCHE undergoing hepatectomy have a poor prognosis. Our results replicate these findings in an independent patient dataset, suggesting that PCHE serves as a prognostic factor in patients undergoing hepatectomy. Furthermore, we found PCHE is prognostic in any individual with HCC, regardless of the treatment they received.

PCHE could be valuable in various clinical scenarios beyond assessing hepatectomy risks. First, it could help risk-stratify patients with HCC undergoing other types of procedures or treatments, such as locoregional or systemic drug therapies. Second, in liver transplantation candidates, PCHE levels might be used in assessing the urgency of transplantation due to mortality risk, and should be studied for post-transplantation outcomes. For instance, it could potentially be used to risk-stratify patients who fall within the Milan criteria to help determine the urgency of transplantation. Third, PCHE could predict mortality in non-cirrhotic HCC, whereas existing systems like BCLC have shown suboptimal prognostic discrimination in this subgroup.^42^ Our analysis of 44 patients with non-cirrhotic HCC revealed that low PCHE levels were linked to increased mortality. Finally, the PCHE level, in conjunction with other prognostic factors, could facilitate more informed discussions between clinicians and patients about treatment options, risks, and benefits. Patients and their families who receive accurate information about prognosis are significantly more likely to opt for hospice care and less likely to receive aggressive treatments (eg, drug therapy, ICU care) near the end of life, which is associated with improved quality of life and reduced unnecessary interventions.^43^,^44^ In summary, PCHE may serve as a valuable biomarker for risk stratification and management of patients with HCC of diverse etiologies.

PCHE presents a potentially low-cost method for estimating prognosis in HCC. A study compared the costs in euros of using PCHE as an inflammatory marker and found that PCHE was significantly less expensive in Greece, India, South Africa, Kenya, Slovakia, and Armenia than procalcitonin (PCT).^45^ Furthermore, in Greece, Kenya, and Armenia, PCHE was also significantly less expensive than C-reactive protein (CRP).^45^ This approach could potentially be applied worldwide, even in countries with limited resources. However, additional information regarding costs in the United States and other nations is needed.

Our study has several limitations. Due to the retrospective design, we can only show associations and not draw conclusions about causality. In addition, the lack of diversity in our study population, which primarily consisted of individuals who self-identified as White in a single tertiary center, limits the generalizability of our findings. Despite the small sample size of patients who underwent hepatic resection, we replicated the outcome in an independent patient dataset. Future studies with a prospective design and a diverse population are needed to validate our findings and gather information regarding outcomes with different treatment interventions according to PCHE levels. However, a key strength of this study is that it is the first study to analyze PCHE in heterogeneous patients with HCC undergoing a variety of treatment modalities. In addition, it is the first study to evaluate the survival rate associated with PCHE compared with existing clinical scoring systems, finding that PCHE adds independent value to these systems.

## CONCLUSIONS

PCHE serves as an independent prognostic biomarker for OS in HCC individuals. Lower PCHE levels are associated with high mortality and poor outcomes. The findings suggest that monitoring PCHE levels could enhance risk stratification and treatment decisions for HCC patients, providing a valuable tool for clinicians seeking to improve patient management and outcomes. PCHE enhances the performance of the existing scoring systems, especially the MESIAH score. By refining and expanding prognostic tools in HCC, PCHE could significantly improve prognostic performance for clinical decision-making.

## Supplementary Material
