# Supplementary material for: Low serum pseudocholinesterase levels are associated with mortality in patients with hepatocellular carcinoma
Source: Hepatol Commun. 2026 Jan 5;10(1):e0879. doi: 10.1097/HC9.0000000000000879 (PMC12772465; doi:10.1097/HC9.0000000000000879)

**Supplementary Figures**

Supplementary Figure 1. Associations of liver function scores with overall survival. Panel A: MESIAH. Panel B: ALBI. Panel C: MELD version 3.0. Panel D: BCLC. Logrank tests p<0.001 for each.

A


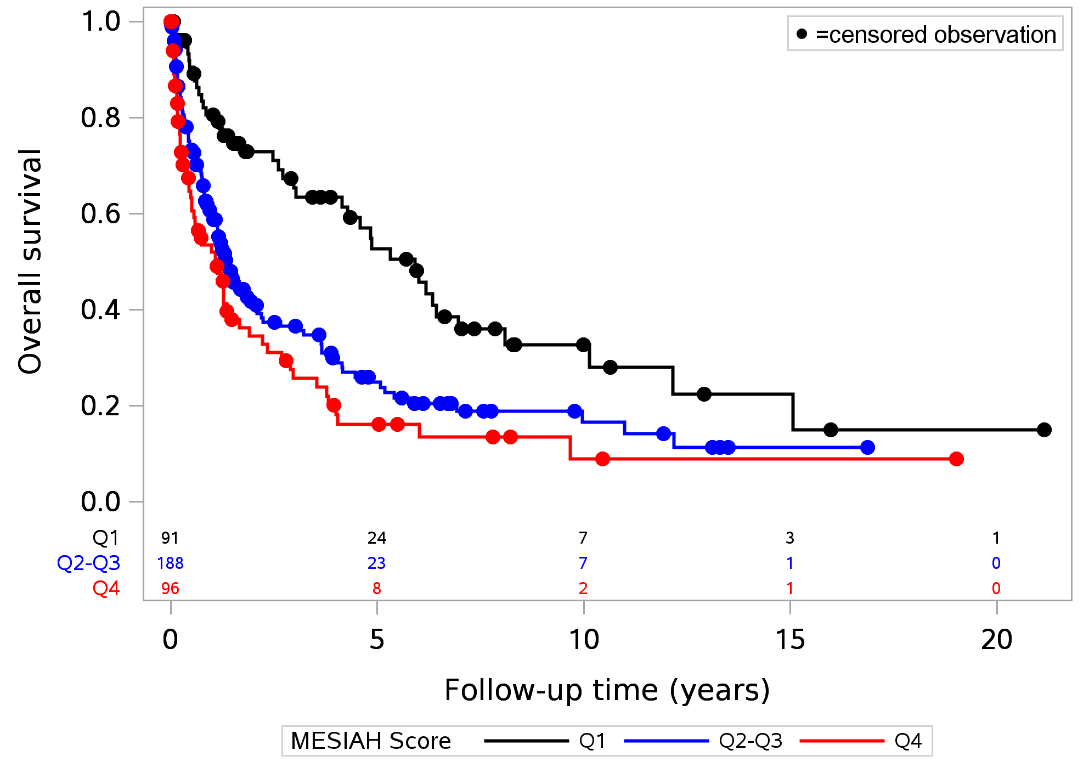


B


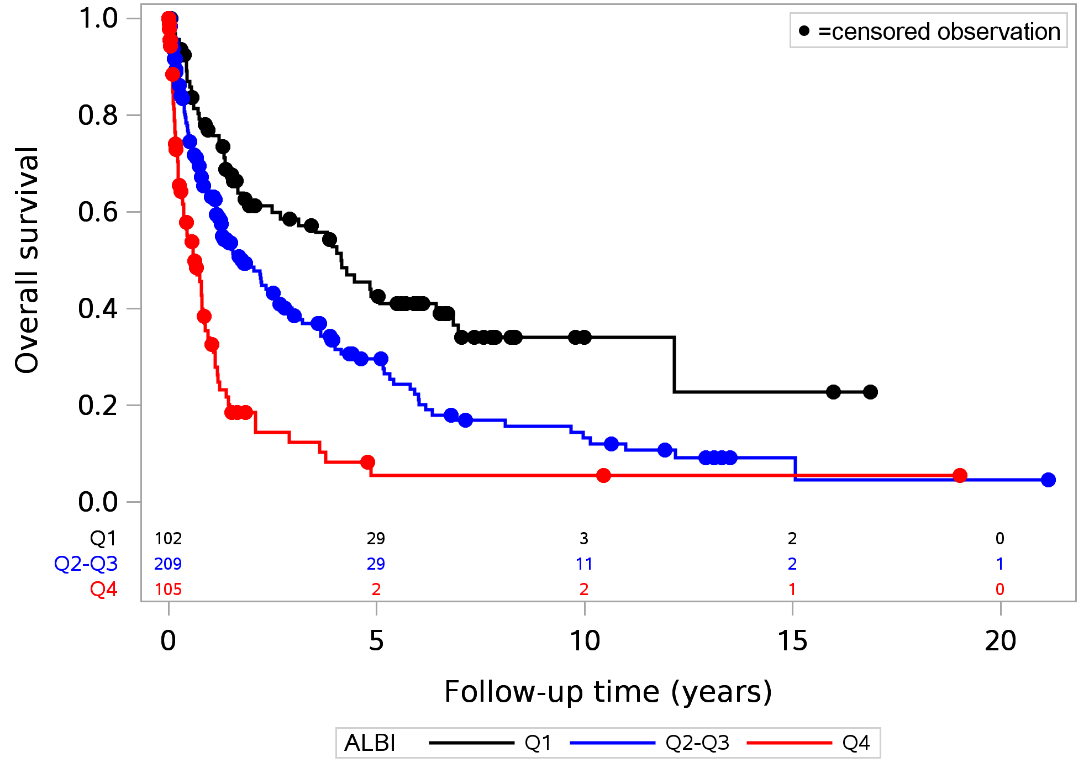


C


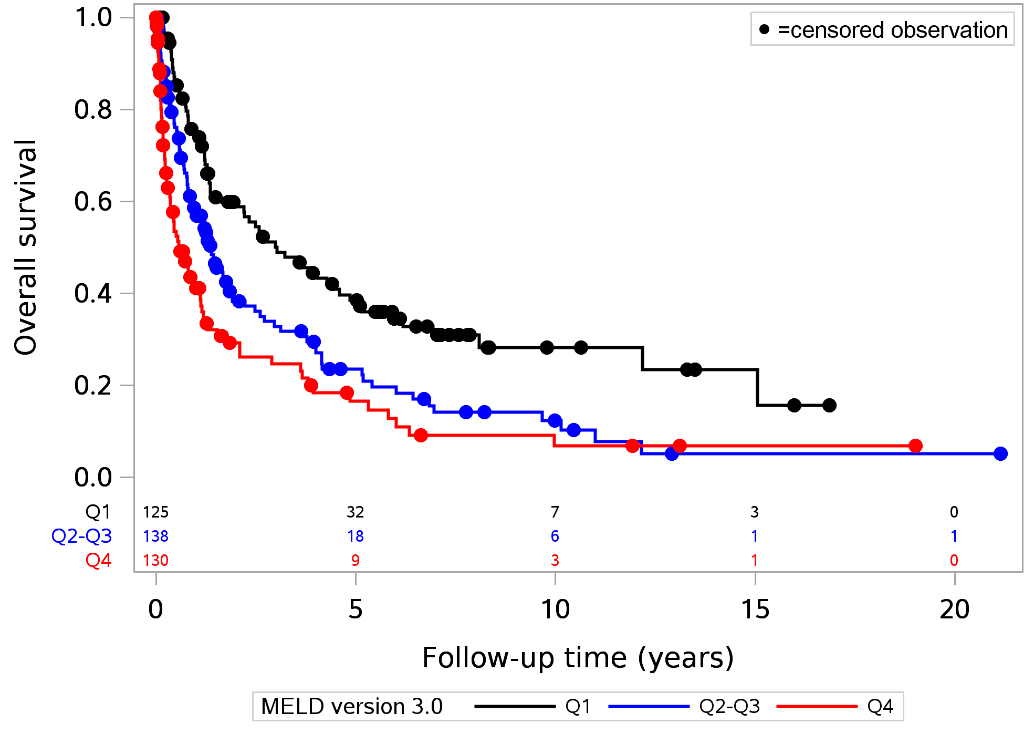


D


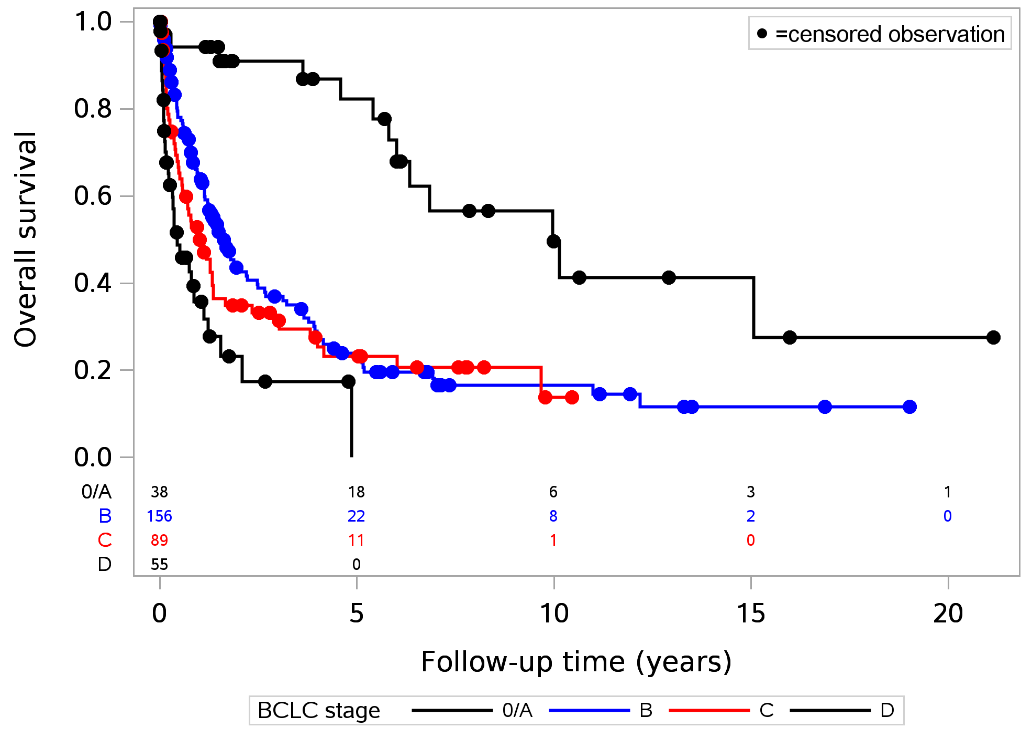


Supplemental Figure 2. Associations of pseudocholinesterase values with other liver function scores. Panel A: scatterplot matrix with MESIAH, ALBI and MELD version 3.0. Panel B: boxplots per level of BCLC stage. Correlation values with pseudocholinesterase: MESIAH, -0.15; ALBI, -0.65; MELD 3.0, -0.41; BCLC, -0.31.

A


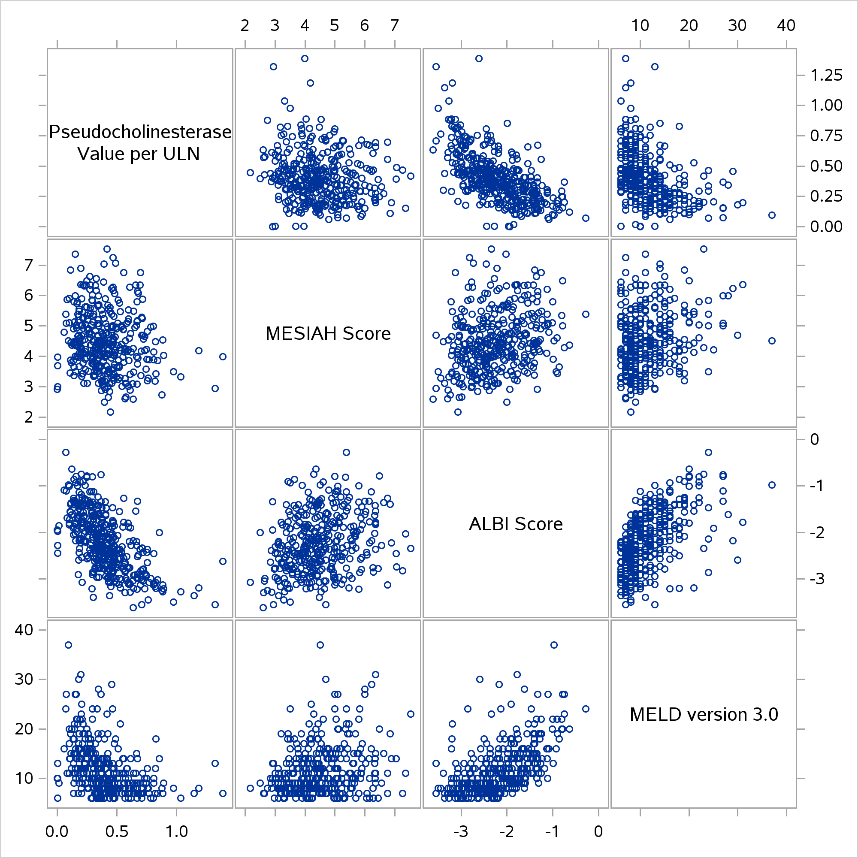


B


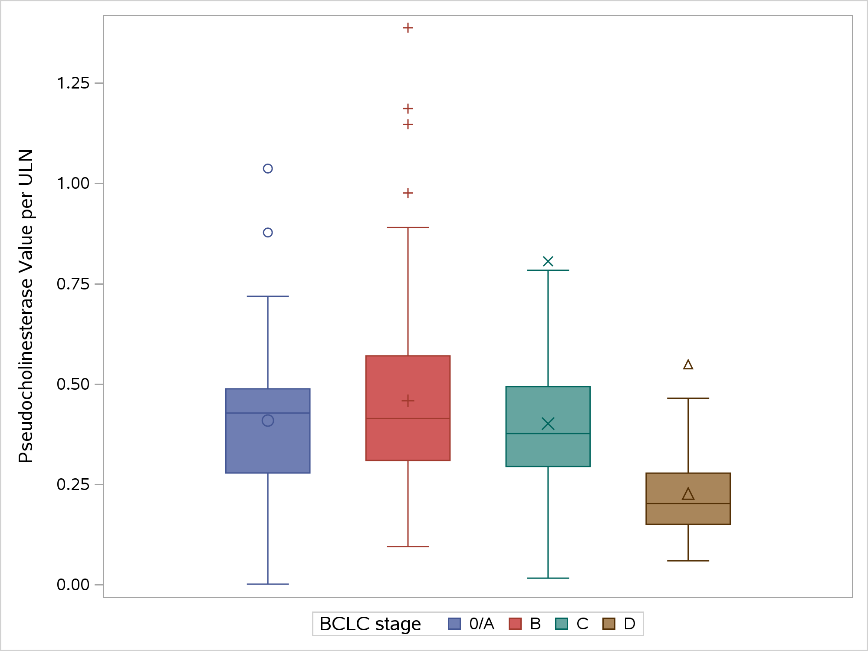


Supplemental Figure 3. Associations of liver function scores with three-month survival, represented as ROC curves and areas under the curve. Panel A: MESIAH. Panel B: ALBI. Panel C: MELD version 3.0. Panel D: BCLC.

A


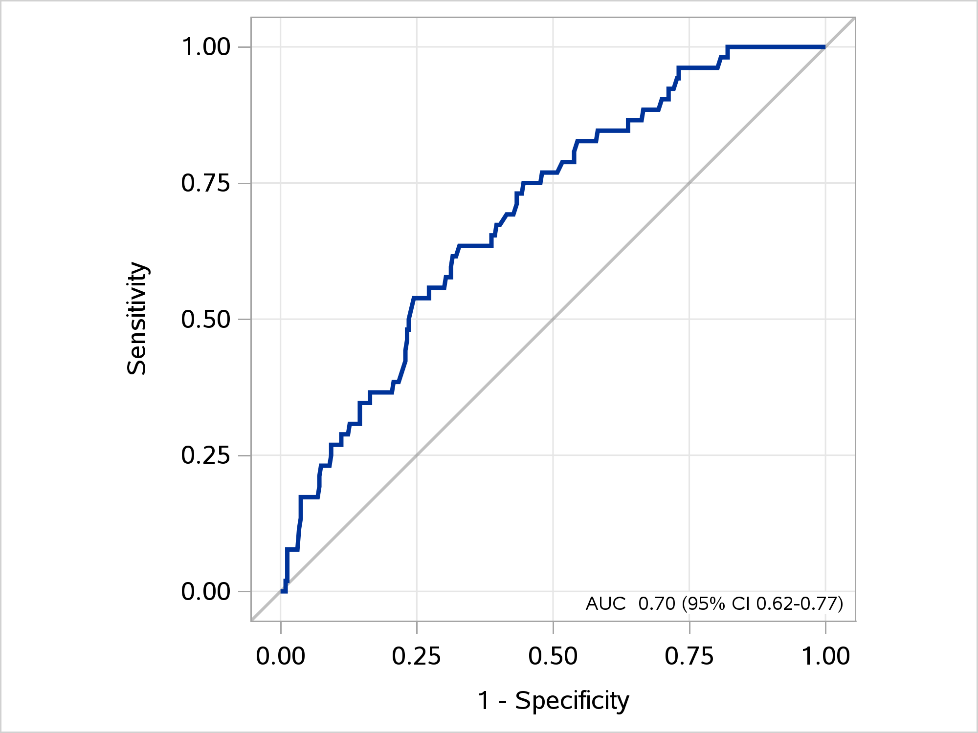


B


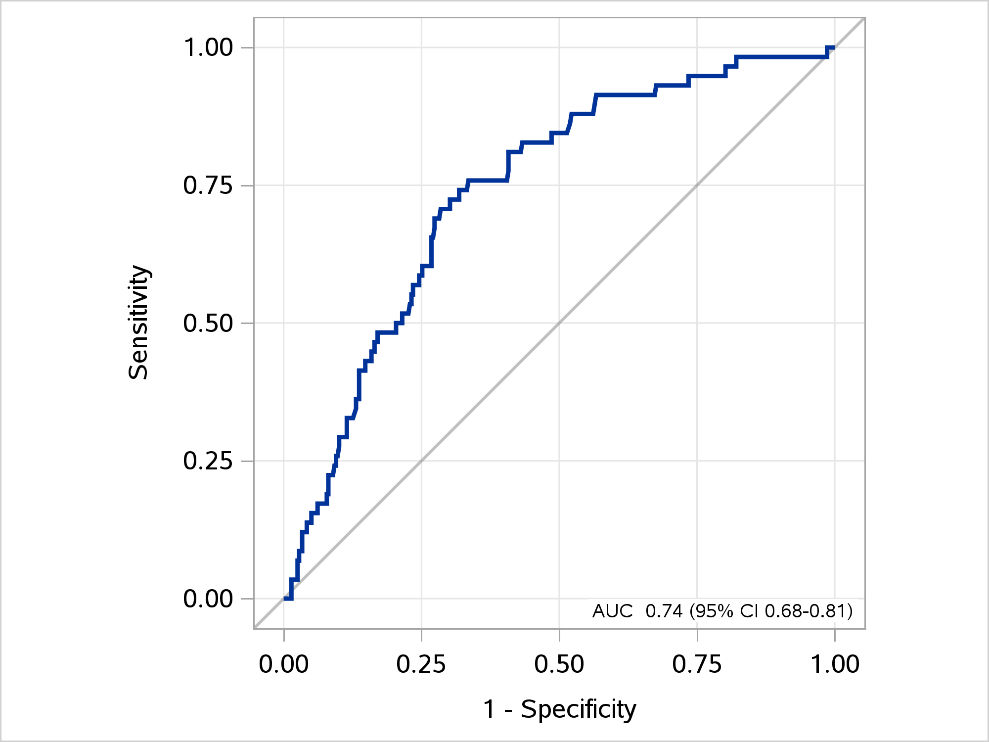


C


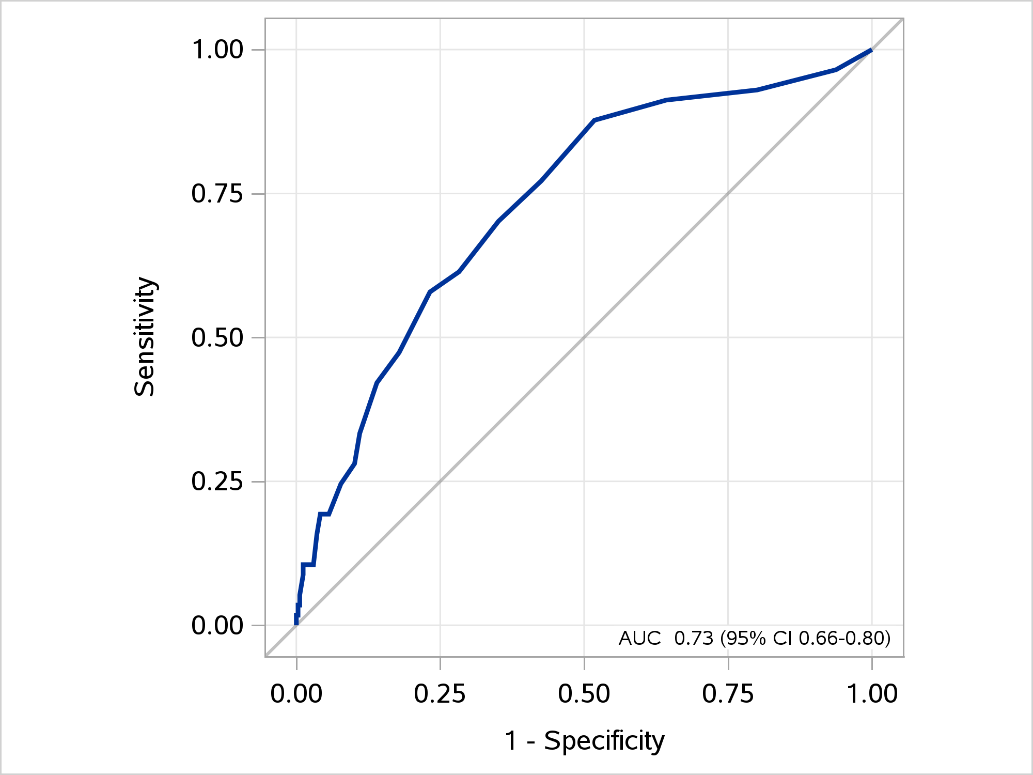


D


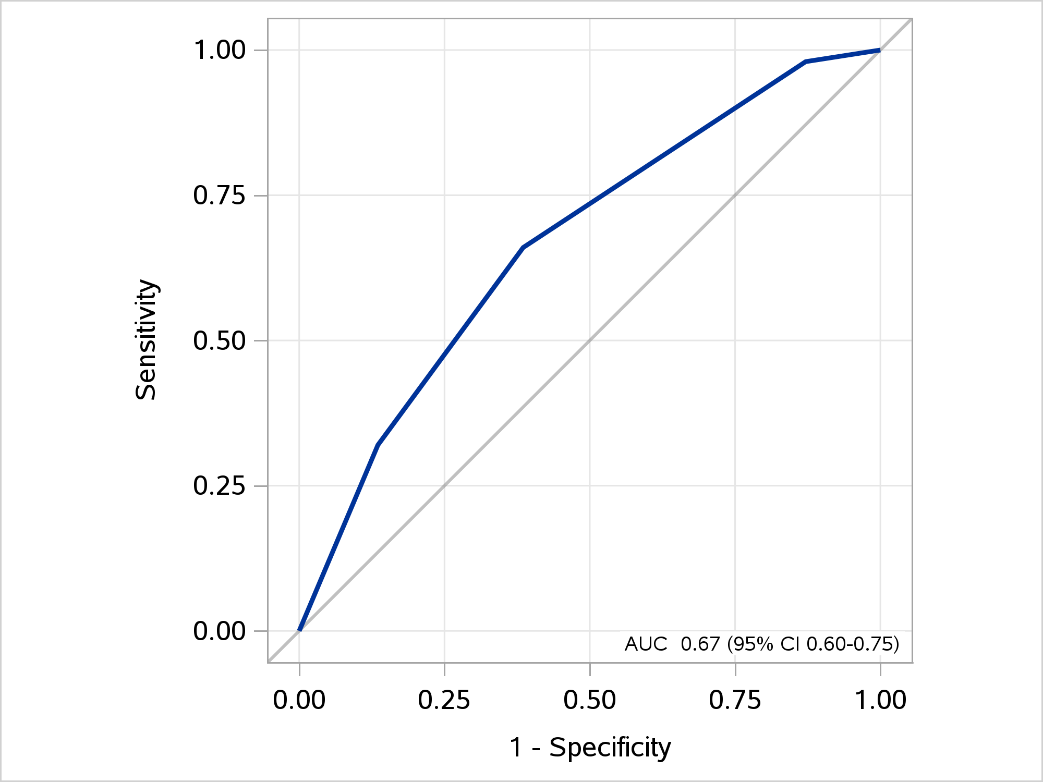

Supplement: Supplementary file 1 [file hc9-10-e0879-s001.docx]
