# Supplementary material for: Low serum pseudocholinesterase levels are associated with mortality in patients with hepatocellular carcinoma
Source: Hepatol Commun. 2026 Jan 5;10(1):e0879. doi: 10.1097/HC9.0000000000000879 (PMC12772465; doi:10.1097/HC9.0000000000000879)
Supplement: Supplementary file 2 [file hc9-10-e0879-s002.docx]

**Supplemental Table**

Supplemental Table 1. Proportion of patients who died within three months of the pseudocholinesterase lab tests, by deciles of pseudocholinesterase values.

|  | | | | |
| --- | --- | --- | --- | --- |
|  | Patient died within three months | |  | |
|  | No (N=362) | Yes (N=58) | Total (N=420) | P-value |
| **Pseudocholinesterase per upper limit of normal**, n (%) |  |  |  | <.001^1^ |
| Decile 1 | 26 (63.4%) | 15 (36.6%) | 41 (9.8%) |  |
| Decile 2 | 31 (72.1%) | 12 (27.9%) | 43 (10.2%) |  |
| Decile 3 | 32 (76.2%) | 10 (23.8%) | 42 (10.0%) |  |
| Decile 4 | 36 (87.8%) | 5 (12.2%) | 41 (9.8%) |  |
| Decile 5 | 37 (86.0%) | 6 (14.0%) | 43 (10.2%) |  |
| Decile 6 | 41 (97.6%) | 1 (2.4%) | 42 (10.0%) |  |
| Decile 7 | 41 (97.6%) | 1 (2.4%) | 42 (10.0%) |  |
| Decile 8 | 40 (95.2%) | 2 (4.8%) | 42 (10.0%) |  |
| Decile 9 | 37 (88.1%) | 5 (11.9%) | 42 (10.0%) |  |
| Decile 10 | 41 (97.6%) | 1 (2.4%) | 42 (10.0%) |  |
| 1. Chi-square test | | | | |
